# Effects of quercetin on polycystic ovary syndrome in animal models: a systematic review and meta-analysis

**DOI:** 10.1186/s12958-024-01220-y

**Published:** 2024-04-18

**Authors:** Pingping Su, Chao Chen, Liang Pang, Kai Wu, Yun Sun

**Affiliations:** 1 The Third School of Clinical Medicine, Zhejiang Chinese Medical University, Hangzhou, China; 2 The Second Affiliated Hospital, Yuying Children’s Hospital of Wenzhou Medical University, Wenzhou, China; 3 Wenzhou TCM Hospital of Zhejiang Chinese Medical University, Wenzhou, China

**Keywords:** Metabolism, Endocrine, GLUT4 gene, Oxidative stress, Quercetin, PCOS

## Abstract

**Background:**

Metformin is an insulin sensitizer that is widely used for the treatment of insulin resistance in polycystic ovary syndrome patients. However, metformin can cause gastrointestinal side effects.

**Purpose:**

This study showed that the effects of quercetin are comparable to those of metformin. Therefore, this study aimed to systematically evaluate the efficacy of quercetin in treating PCOS.

**Methods:**

The present systematic search of the Chinese National Knowledge Infrastructure (CNKI), Wanfang Data Information Site, Chinese Scientific Journals Database (VIP), SinoMed, Web of Science, and PubMed databases was performed from inception until February 2024. The methodological quality was then assessed by SYRCLE’s risk of bias tool, and the data were analyzed by RevMan 5.3 software.

**Results:**

Ten studies were included in the meta-analysis. Compared with those in the model group, quercetin in the PCOS group had significant effects on reducing fasting insulin serum (FIS) levels (*P* = 0.0004), fasting blood glucose (FBG) levels (*P* = 0.01), HOMA-IR levels (*P* < 0.00001), cholesterol levels (*P* < 0.0001), triglyceride levels (*P* = 0.001), testosterone (T) levels (*P* < 0.00001), luteinizing hormone (LH) levels (*P* = 0.0003), the luteinizing hormone/follicle stimulating hormone (LH/FSH) ratio (*P* = 0.01), vascular endothelial growth factor (VEGF) levels (*P* < 0.00001), malondialdehyde (MDA) levels (*P* = 0.03), superoxide dismutase (SOD) levels (*P* = 0.01) and GLUT4 mRNA expression (*P* < 0.00001).

**Conclusion:**

This meta-analysis suggested that quercetin has positive effects on PCOS treatment. Quercetin can systematically reduce insulin, blood glucose, cholesterol, and triglyceride levels in metabolic pathways. In the endocrine pathway, quercetin can regulate the function of the pituitary-ovarian axis, reduce testosterone and luteinizing hormone (LH) levels, and lower the ratio of LH to follicle-stimulating hormone (FSH). Quercetin can regulate the expression of the GLUT4 gene and has antioxidative effects at the molecular level.

**Supplementary Information:**

The online version contains supplementary material available at 10.1186/s12958-024-01220-y.

## Introduction

Quercetin, a natural compound, is a flavonoid compound. Its molecular formula is C15H10O7, and its chemical name is 4 h-1-benzopyran-4-one, 2-(3,4-dihydroxy phenyl), 3,5,7trihydroxy-flavone. Quercetin is an effective ingredient in dietary polyphenols and is considered the flavonoid with the highest intake [1, 2]. Quercetin is widely present in many fruits and vegetables, especially in berries, apples, oranges, red grapes, tomatoes, buckwheat, onions, and broccoli [3]. Traditionally, quercetin has been used for the prevention and treatment of various diseases, such as diabetic nephropathy [4], neurological and neurodegenerative diseases [5], ischemic brain injury [6], and cardiovascular diseases [7]. Modern pharmacological research has shown that quercetin has multiple functions, such as reducing oxidative stress [8], reducing inflammation [9], protecting nerves [10], inhibiting platelet aggregation [11], relaxing vascular smooth muscle [12], and inhibiting cancer development [13]. These studies indicate that quercetin plays an important role in protecting health and preventing diseases. Quercetin has many therapeutic effects on the pathological process of polycystic ovary syndrome by affecting polycystic ovary syndrome through different targets and pathways, which makes it a potential drug for treating this disease.

**Fig. 1:**
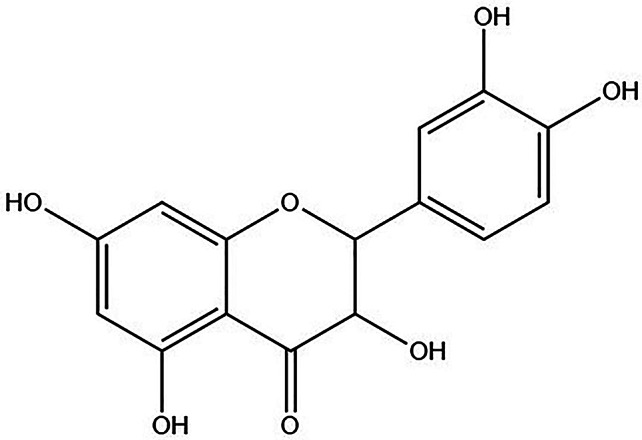
Quercetin chemical formula

In recent years, researchers have conducted extensive research on the therapeutic potential of quercetin in treating polycystic ovary syndrome, including in animal models and clinical trials. However, current reviews on the treatment of polycystic ovary syndrome with quercetin have focused mainly on its efficacy and characteristics, and explorations and summaries of its potential mechanisms are lacking. Therefore, this study aimed to integrate and analyze these animal experiments, systematically evaluate the therapeutic effects, explore the relevant mechanisms, and further provide scientific references for the clinical application of quercetin in PCOS (Fig. 1).

## Materials and methods

### Data sources and searches

We selected relevant studies published by February 2024 that were not limited by language by searching databases including the Chinese National Knowledge Infrastructure (CNKI), Wanfang Data Information Site, Chinese Scientific Journals Database (VIP), SinoMed, Cochrane Library, Web of Science, and PubMed. The search terms used were (“Quercetin”) AND (“Polycystic Ovarian Syndrome” OR “Stein-Leventhal Syndrome” “Sclerocystic Ovarian Degeneration” OR “Sclerocystic Ovary Syndrome” OR “PCOS”). Comprehensive and systematic retrieval was conducted according to different database situations. The obtained literature was imported into NoteExpress software.

### Inclusion criteria

#### Research subjects

The animal models met the standards for PCOS treatment.

#### Intervention

The experimental group was treated with quercetin, and the control group did not receive any treatment. The difference in intervention measures between the experimental and control groups should only be whether quercetin treatment was used.

#### Efficacy evaluation index

FIS, FBG, HOMA-IR, cholesterol, TG, LH, LH/FSH, T, VEGF, MDA, SOD, GLUT4 mRNA.

#### Research design

There were no restrictions on language or time.

### Exclusion criteria

(1) Review, case, dissertation, catalog, and other informal journal; (2) Clinical and in vitro research; (3) Duplicated study and unrelated literature; (4) Nonpolycystic ovarian syndrome animal model; (5) Drug combination; (6) The original text is lost or the original data are missing; (7) The outcome indicators could not be extracted or merged.

### Data extraction

Two researchers who received the same training used NoteExpress software to screen the literature independently, and any contradictions were addressed by a third researcher. The main data extracted included the following: (1) general information, including the literature title, first author’s name, year of publication, country, animal species, age, weight, model, modeling material, and method of administration; (2) intervention measures, including drug name, dosage and time; and (3) outcome. For the studies that only reported data in the form of images, we extracted the data from the images through GetData Graph Digitizer software.

### Quality assessment

The SYRCLE risk of bias tool was used by two researchers to make low-risk, high-risk and unclear judgments for each entry according to the appropriate criteria. The statistical software RevMan 5.3 was used to create risk of bias plots. The standard lists included sequence generation (selection bias), baseline characteristic (selection bias), allocation concealment (selection bias), random housing (performance bias), blinding (performance bias), random outcome assessment (detection bias), blinding (detection bias), incomplete outcome data (attrition bias), selective outcome reporting (reporting bias), and other sources of bias (other). Any disagreements were resolved by discussion with another researcher.

### Statistical analysis

The statistical software RevMan 5.3 was used to analyze the collected data. The mean difference (MD) or standardized mean difference (SMD) was used for continuous variables to calculate their 95% confidence intervals (95% CIs). When the heterogeneity test results were *P* > 0.05 and I^2^ < 50%, heterogeneity was considered low or nonexistent, and fixed effect models were used. When the heterogeneity test results were *P* < 0.05 and I^2^ > 50%, which represented high heterogeneity, random effect models were used. For the literature with excessive heterogeneity, further analysis, such as subgroup analysis, was performed to determine the source of heterogeneity according to the actual situation.

## Results

### Study inclusion

**Fig. 2:**
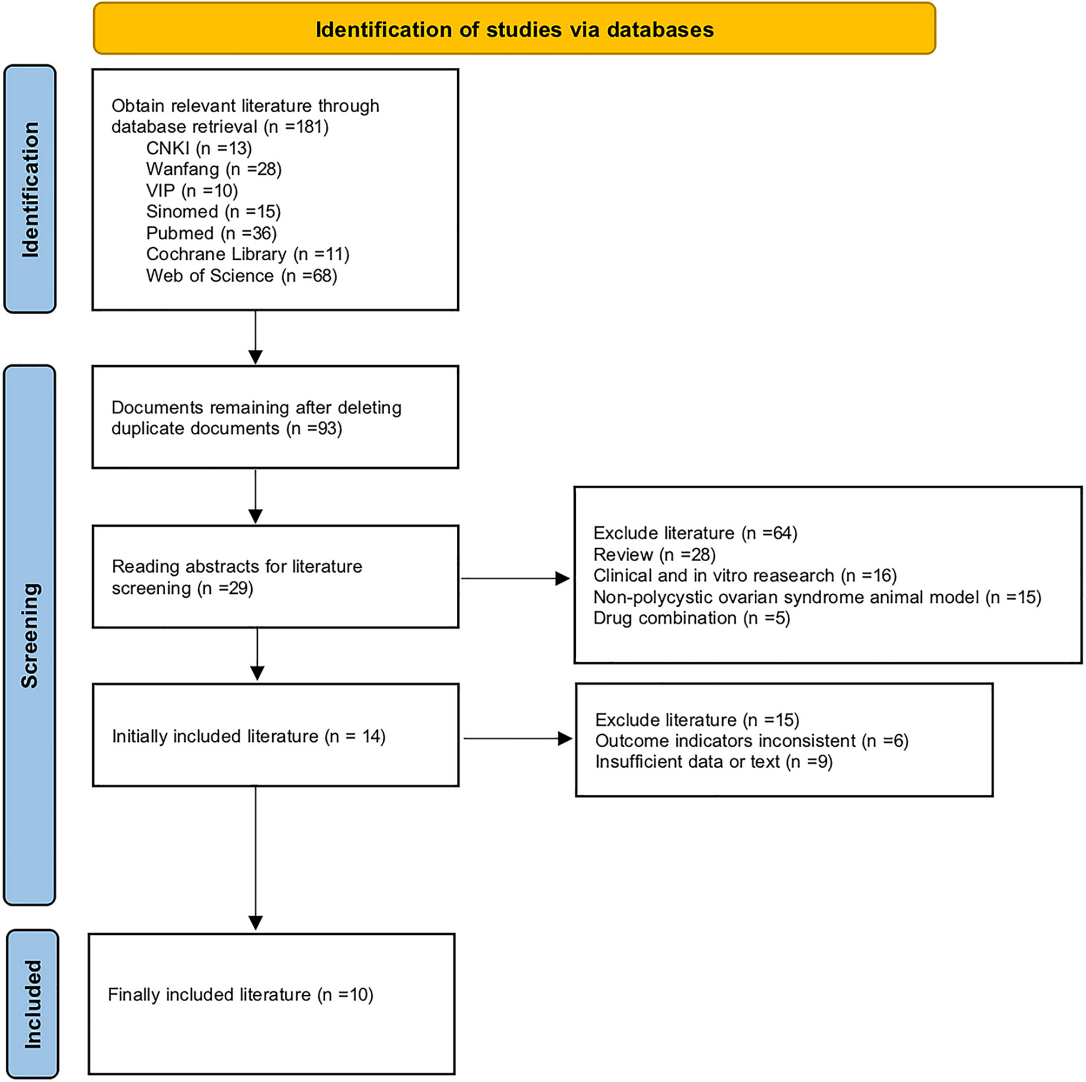
Literature search flow chart

This study was conducted in strict accordance with the guidelines of the System Evaluation and Meta-Analysis (PRISMA) and the Cochrane Collaboration. We identified a total of 181 studies. Ninety-three studies remained after excluding replication literature, and 29 studies remained after excluding reviews, clinical and in vitro research, nonpolycystic ovarian syndrome animal models, and drug combinations. Eventually, 10 studies were included in the final meta-analysis (Fig. 2). The basic characteristics of the included studies are shown in Tables 1 and 2.

**Table 1:** Basic characteristics of the included studies

Study	Country	Animal characteristics	Model	Modeling material	Method of administration	Dose of quercetin	T	E	C	Outcome measures
Neisy A.,2019 [14]	Iran	female SD rats (180 g)	PCOS	DHEA	Be injected with 6 mg/100 g/d DHEA dissolved in 0.2 ml sesame oil for 21 days to induce PCOS	15 mg/kg Q dissolved in 0.5 mL 10% ethanol	30d	Q	Didn’t receive any treatment	①②③⑫
Shah M.Z.U.H.,2023 [15]	India	mature parkes strain mice (18–21 g)	PCOS	LETZ	Be given 6 mg/kg LETZ for 21 days to induce PCOS	125 mg/kg Q	45d	Q	Didn’t receive any treatment	④⑤⑥⑧⑨
Zheng S.Y.,2022 [16]	China	female SD rats (21-day-old)	PCOS	DHEA	Be injected with 6 mg/100 g/d DHEA dissolved in 0.2 ml sesame oil for 20 days to induce PCOS	100 mg/kg Q dissolved in 2 ml 1% sodium carboxymethyl cellulose solution	28d	Q	2 ml 1% sodium carboxymethylcellulose solution	③⑥⑦⑧
Wang Z.Z.,2017 [17]	China	female Wistar rats (21-day-old)	PCOS	DHEA	Be injected with 6 mg/100 g/d DHEA dissolved in 0.2 ml sesame oil for 20 days to induce PCOS	2 mL of Q solution (100 mg/kg)	28d	Q	2 mL of 1% CMC	②③
Olaniyan O.T.,2020 [8]	Nigeria	prepubertal female Wistar rats (21-day-old) (18 ± 1.58 g)	PCOS	DHEA	Be injected with 6 mg/100 g/d DHEA dissolved in 0.2 ml corn oil to induce PCOS	100 mg/kg Q	15d	Q	Didn’t receive any treatment	⑨⑩⑪
Mahmoud A.A.,2022 [18]	Egypt	prepubertal female Wistar rats (23-day-old) (26.45 ± 0.06 g)	PCOS	DHEA	Be injected wtih 60 mg/kg DHEA dissolved in 1 ml sesameoil for 41 days to induce PCOS	25 mg/kg Q dissolved in 0.05 ml saline	28d	Q	saline	⑥⑦⑧
Mihanfar A.,2021 [19]	Iran	female Wistar rats (42-day-old)	PCOS	LETZ	Be given 1 mg/kg LETZ dissolved in CMC 0.5% for 21 days to induce PCOS	100 mg/kg Q dissolved in CMC 0.5%	30d	Q	Didn’t receive any treatment	①②③④⑤⑥
Jahan S.,2018 [20]	Pakistan	adult female SD rats (60-70-day-old) (180 ± 10 g)	PCOS	LETZ	Be given 1 mg/kg LETZ dissolved in 0.5% CMC to induce PCOS	30 mg/kg Q	22d	Q	Didn’t receive any treatment	④⑤⑪
Jiang X.J.,2022 [21]	China	SPF female SD rats (21-23-day-old)	PCOS	LETZ	Be given 1 mg/kg LETZ dissolved in 0.4 ml CMC to induce PCOS	30 mg/kg Q dissolved in 0.5 mL 10% ethanol	28d	Q	0.5 mL 10% ethanol	①②⑫
Yang Z.,2021 [22]	China	minor female SD rats (21-day-old) (80–90 g)	PCOS	DHEA	Be injected with 6 mg/100 g/d DHEA dissolved in 0.2 ml olive oil for 20 days to induce PCOS	100 mg/kg Q dissolved in 2 ml CMC	1d	Q	2 mL CMC	⑥⑦⑩⑪

*Abbreviations* SD: Sprague–Dawley; Q: quercetin; PCOS: polycystic ovary syndrome; DHEA: dehydroepiandrosterone; LETZ: letrozole; CMC: carboxy methylcellulose; T: time; E: experimental group; C: control group

*Notes* ① Fasting insulin serum levels; ② fasting blood glucose levels; ③ HOMA-IR; ④ cholesterol levels; ⑤ triglyceride levels; ⑥ testosterone levels; ⑦ luteinizing hormone levels; ⑧ luteinizing hormone/follicle stimulating hormone ratio; ⑨ vascular endothelial growth factor levels; ⑩ malondialdehyde levels; ⑪ superoxide dismutase levels; and ⑫ the expression of GLUT4 mRNA

### Risk of bias assessment

**Fig. 3:**
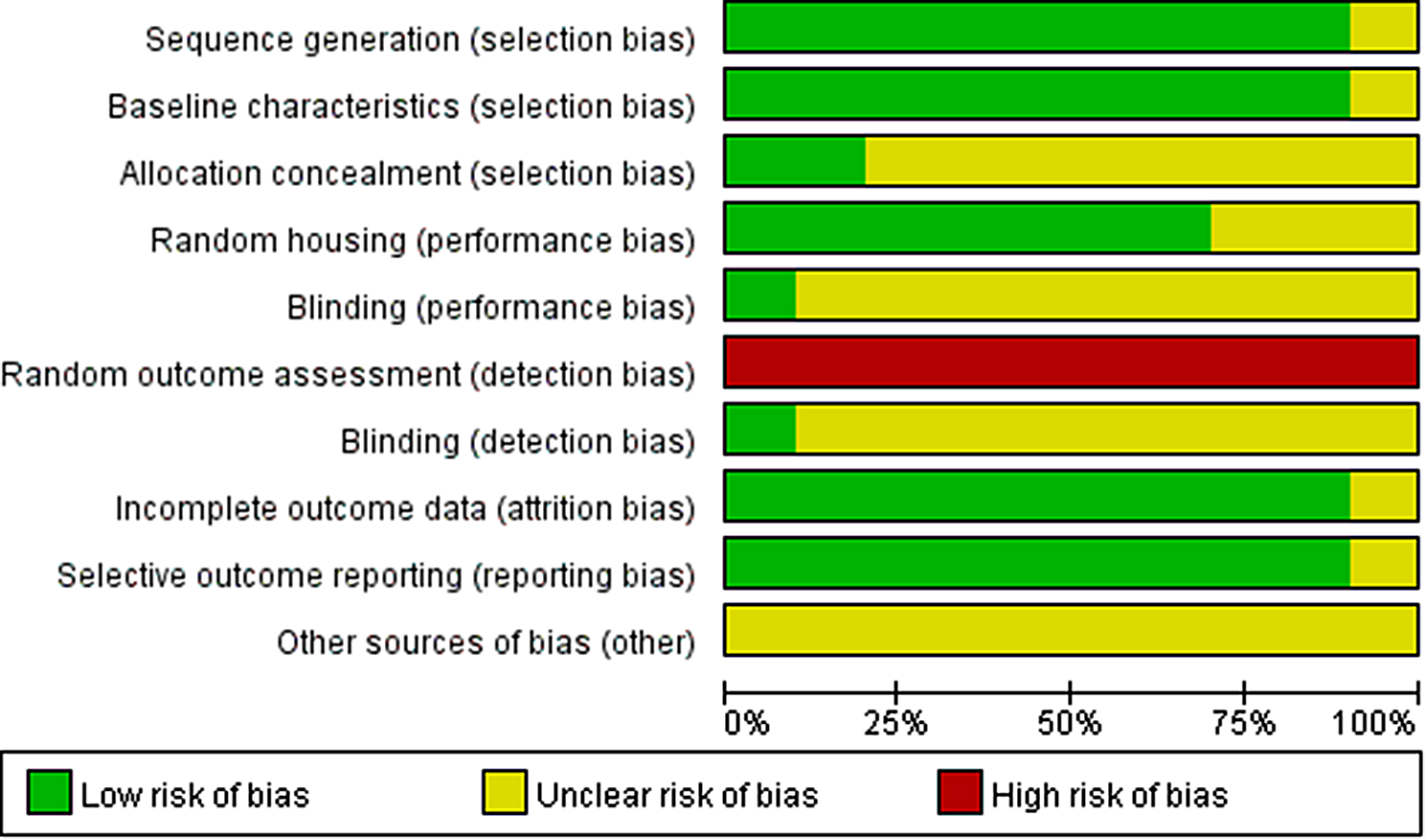
Risk of bias summary. A summary table of review authors’ judgments for each risk of bias item for each study

**Fig. 4:**
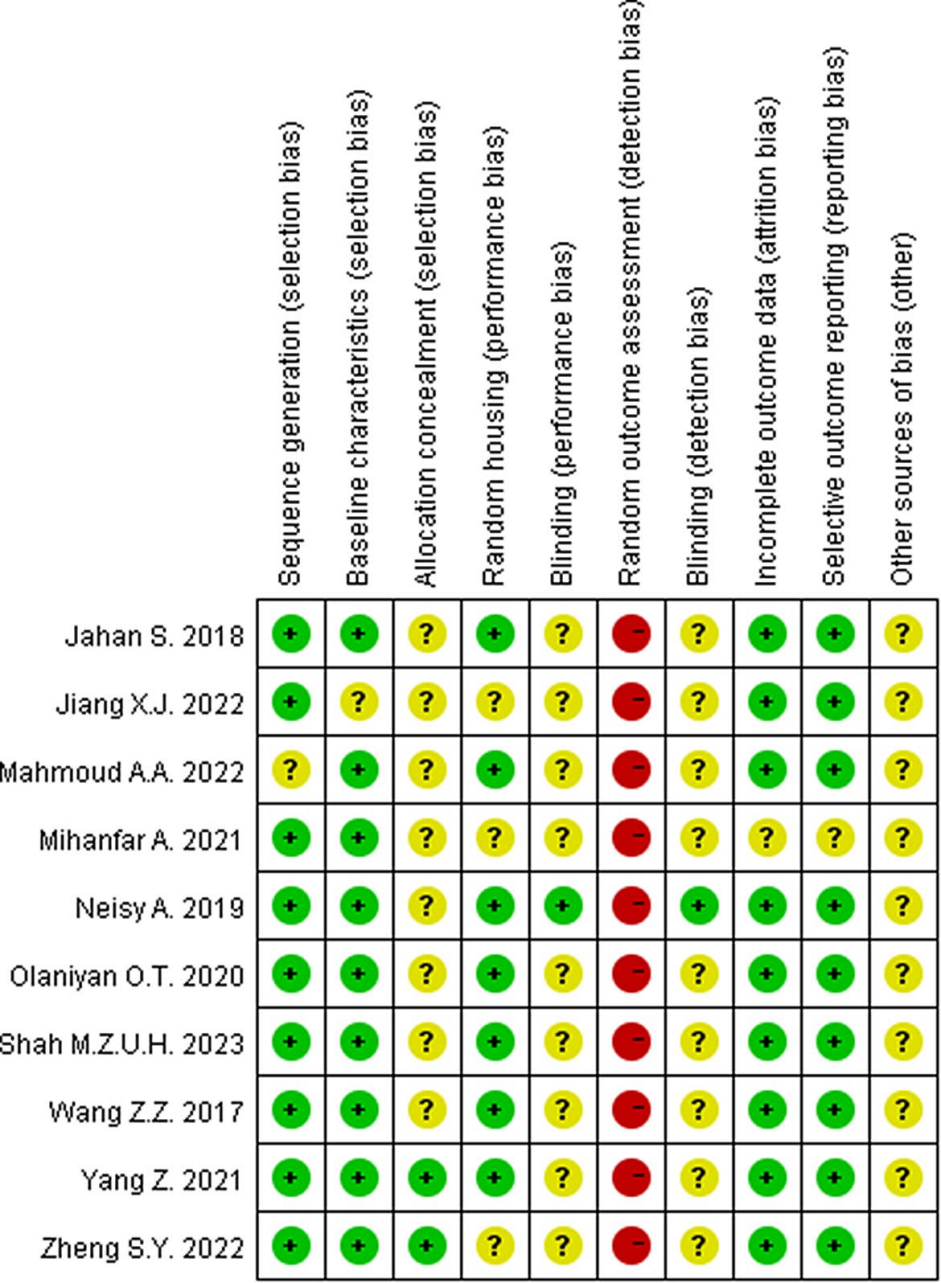
Risk of bias graph. A plot of the distribution of review authors’ judgments across studies for each risk of bias item. *Note* “+ " represents low risk; “? “represents unclear risk; “-” represents high risk

We assessed the quality of the literature using SYRCLE’s risk of bias tool, and the data were analyzed with RevMan 5.3 software (Fig. 3 and Fig. 4).

### Meta-analysis

#### Fasting insulin serum

Three studies [14, 19, 21] reported FIS levels. There was heterogeneity (*P* ≤ 0.00001, I^2^ = 94%), and a random effects model was adopted. The results showed that quercetin could effectively reduce FIS levels (MD=-9.22, 95% CI=[-15.41, -3.02], *P* = 0.004), and the difference was statistically significant (Table 2).

#### Fasting blood glucose

Three studies [14, 17, 19] reported FBG levels. There was heterogeneity (*P* = 0.007, I^2^ = 80%), and the random effect model was adopted. The results showed that quercetin could effectively reduce FBG levels (MD=-2.57, 95% CI=[-4.60, -0.55], *P* = 0.01), and the difference was statistically significant (Table 2).

#### HOMA-IR

Four studies [14, 16, 17, 19] reported HOMA-IR. There was heterogeneity (*P* < 0.00001, I^2^ = 99%), and the random effect model was adopted. The results showed that quercetin could effectively reduce HOMA-IR (MD=-4.13, 95% CI=[-5.82, -2.44], *P* < 0.00001), and the difference was statistically significant (Table 2).

#### Cholesterol

Three studies [15, 19, 20] reported cholesterol levels. There was heterogeneity (*P* = 0.08, I^2^ = 61%), and the random effect model was adopted. The results showed that quercetin could effectively reduce cholesterol levels (MD=-6.82, 95% CI=[-10.21, -3.44], *P* < 0.0001), and the difference was statistically significant (Table 2).

#### TG levels

Three studies [15, 19, 20] reported TG levels. There was heterogeneity (*P* = 0.02, I^2^ = 73%), and the random effect model was adopted. The results showed that quercetin could effectively reduce TG levels (MD=-7.33, 95% CI=[-11.73, -2.93], *P* = 0.001), and the difference was statistically significant (Table 2).

#### Testosterone

Four studies [15, 16, 18, 19] reported T levels. There was heterogeneity (*P* < 0.00001, I^2^ = 90%), and the random effect model was adopted. The results showed that quercetin could effectively reduce T levels (MD=-2.02, 95% CI=[-2.79, -1.26], *P* < 0.00001), and the difference was statistically significant (Table 2).

#### Luteinizing hormones

Two studies [16, 18] reported LH levels. There was heterogeneity (*P* < 0.0001, I^2^ = 94%), and the random effect model was adopted. The results showed that quercetin could effectively reduce LH levels (MD=-9.23, 95% CI=[-14.20, -4.26], *P* = 0.0003), and the difference was statistically significant (Table 2).

#### Luteinizing Hormone/Follicle stimulating hormone

Three studies [15, 16, 18] reported the LH/FSH ratio. There was heterogeneity (*P* < 0.00001, I^2^ = 98%), and the random effect model was adopted. Quercetin effectively reduced the LH/FSH ratio (MD=-0.94, 95% CI=[-1.70, -0.19], *P* = 0.01), and the difference was statistically significant (Table 2).

#### Vascular endothelial growth factor

Two studies [8, 15] reported VEGF levels. There was heterogeneity (*P* = 0.01, I^2^ = 85%), and the random effect model was adopted. The results showed that quercetin could effectively reduce VEGF levels (MD=-6.69, 95% CI=[-9.14, -4.24], *P* < 0.00001), and the difference was statistically significant (Table 2).

#### Malondialdehyde

Two studies [8, 22] reported MDA levels. There was heterogeneity (*P* = 0.04, I^2^ = 76%), and a random effects model was adopted. The results showed that quercetin could effectively reduce MDA levels (MD=-2.66, 95% CI=[-5.01, -0.31], *P* = 0.03), and the difference was statistically significant (Table 2).

#### Superoxide dismutase

Three studies [8, 20, 22] reported SOD levels. There was heterogeneity (*P* = 0.0006, I^2^ = 86%), and a random effects model was adopted. The results showed that quercetin could effectively reduce SOD levels (MD = 5.50, 95% CI=[1.27, 9.74], *P* = 0.01), and the difference was statistically significant (Table 2).

#### GLUT4 mRNA

Two studies [14, 21] reported the expression of GLUT4 mRNA. There was heterogeneity (*P* = 0.16, I^2^ = 49%), and the fixed effect model was adopted. The results showed that quercetin effectively reduced the expression of GLUT4 mRNA (MD = 7.26, 95% CI=[5.14, 9.38], *P* < 0.00001), and the difference was statistically significant (Table 2).

**Table 2:** Meta-analysis results for each outcome indicator

Outcome indicator	Heterogeneity test results		Effect models	Meta-analysis results		
	I^2^(%)	*P*		Effect sizes	95%CI	*P*
Fasting Insulin Serum Levels	94	< 0.00001	random	MD=-9.22	[-15.41, -3.02]	0.004
Fasting Blood Glucose Levels	80	0.007	random	SMD=-2.57	[-4.60, -0.55]	0.01
HOMA-IR	99	< 0.00001	random	MD=-4.13	[-5.82, -2.44]	< 0.00001
Cholesterol Levels	61	0.08	random	SMD=-6.82	[-10.21, -3.44]	< 0.0001
Triglycerides Levels	73	0.02	random	SMD=-7.33	[-11.72, -2.93]	0.001
Testosterone Levels	90	< 0.00001	random	MD=-2.02	[-2.79, -1.26]	< 0.00001
Luteinizing Hormone Levels	94	< 0.0001	random	MD=-9.23	[-14.20, -4.26]	0.0003
Luteinizing Hormone/Follicle Stimulating Hormone Ratio	98	< 0.00001	random	MD=-0.94	[-1.70, -0.19]	0.01
Vascular Endothelial Growth Factor Levels	85	0.01	random	MD=-6.69	[-9.14, -4.24]	< 0.00001
Malondialdehyde Levels	76	0.04	random	MD=-2.66	[-5.01, -0.31]	0.03
Superoxide Dismutase Levels	86	0.0006	random	SMD = 5.50	[1.27, 9.74]	0.01
The Expression of GLUT4 mRNA	49	0.16	Fixed	SMD = 7.26	[5.14, 9.38]	< 0.00001

### Sensitivity analysis

#### Fasting insulin serum

**Fig. 5:**
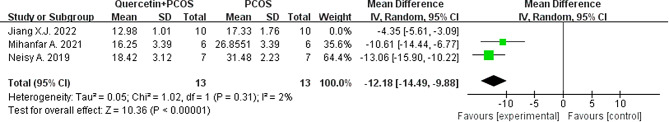
Forest plot of FIS by leave-one out analysis

We conducted a sensitivity analysis of FIS levels by leave-one out analysis. Jiang’s study was the main source of heterogeneity, and I^2^ was directly reduced from 94 to 2%. By reading the original text, we speculated that differences in the sources of the rats included in the studies led to heterogeneity. The rats used in Jiang’s experiment were from China, while in Mihanfar and Neisy’s experiments, the rats were from Iran. However, the results still support that quercetin can reduce FIS levels in PCOS animals (Fig. 5).

#### Fasting blood glucose

**Fig. 6:**
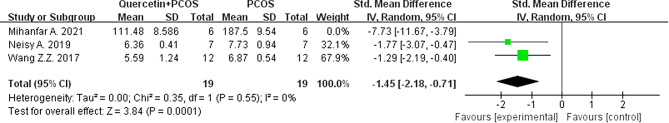
Forest plot of FBG by leave-one out analysis

We conducted a sensitivity analysis of the FBG levels by leave-one out analysis. The study reported by Mihanfar was the main source of heterogeneity, and I^2^ was directly reduced from 80 to 0%. By reading the original text, we speculated that differences in modeling material among the studies led to heterogeneity. In Mihanfar’s experiment, rats were given LETZ to induce PCOS, while rats were injected with DHEA to induce PCOS in Neisy and Wang’s experiments. However, the results still support that quercetin can reduce FBG levels in PCOS animals (Fig. 6).

#### Cholesterol

**Fig. 7:**
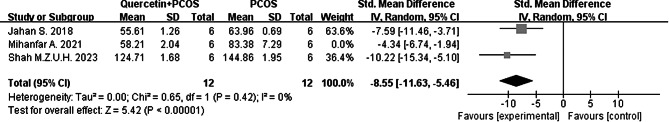
Forest plot of cholesterol by leave-one out analysis

We conducted a sensitivity analysis of cholesterol levels by leave-one out analysis. The study reported by Mihanfar was the main source of heterogeneity, and I^2^ was directly reduced from 61 to 0%. By reading the original text, we speculated that differences in the quercetin source in the studies led to heterogeneity. In Mihanfar’s experiment, the quercetin source was unclear, while in Jahan and Shah’s experiments, quercetin was obtained from Sigma. However, the results still support that quercetin can reduce cholesterol levels in PCOS animals (Fig. 7).

#### Triglycerides

**Fig. 8:**
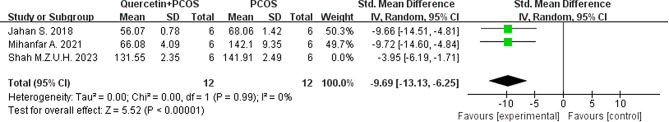
Forest plot of TG by leave-one out analysis

We conducted a sensitivity analysis of TG levels by leave-one out analysis. Shah was the main source of heterogeneity, and I^2^ was directly reduced from 73 to 0%. By reading the original text, we speculated that differences in the animal species in the studies led to heterogeneity. In Shah’s experiment, the animal species was mouse, while in Jahan and Mihanfar’s experiments, the animal species was rat. However, the results still support that quercetin can reduce TG levels in PCOS animals (Fig. 8).

#### Luteinizing Hormone/Follicle stimulating hormone

**Fig. 9:**
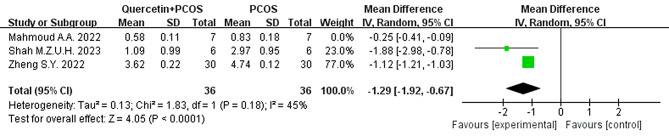
Forest plot of LH/FSH ratio by leave-one out analysis

We conducted a sensitivity analysis of the LH/FSH ratio by leave-one out analysis. The studies reported by Mahmoud were the main source of heterogeneity, and I^2^ was directly reduced from 98 to 45%. By reading the original text, we speculated that differences in the ELISA kit manufacturers used in the studies led to heterogeneity. In Mahmoud’s experiment, the ELISA kits were made in China, while in Shah and Zheng’s experiments, the ELISA kits were made in America. However, the results still support that quercetin can reduce the LH/FSH ratio in PCOS animals (Fig. 9).

#### SOD

**Fig. 10:**
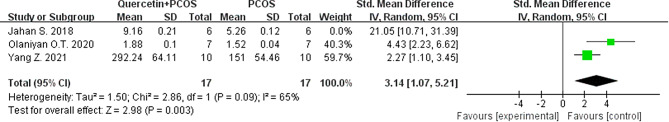
Forest plot of SOD by leave-one out analysis

We conducted a sensitivity analysis of the SOD levels by leave-one out analysis. The studies reported by Jahan were the main source of heterogeneity, and I^2^ was directly reduced from 86 to 65%. By reading the original text, we speculated that differences in the PCOS model inducers used in the studies led to heterogeneity. In Jahan’s experiment, the PCOS model inducer was LETZ, while in Olaniyan and Yang’s experiments, the PCOS model inducer was DHEA. However, the results still support that quercetin can reduce the SOD levels in PCOS animals (Fig. 10).

#### HOMA-IR

**Fig. 11:**
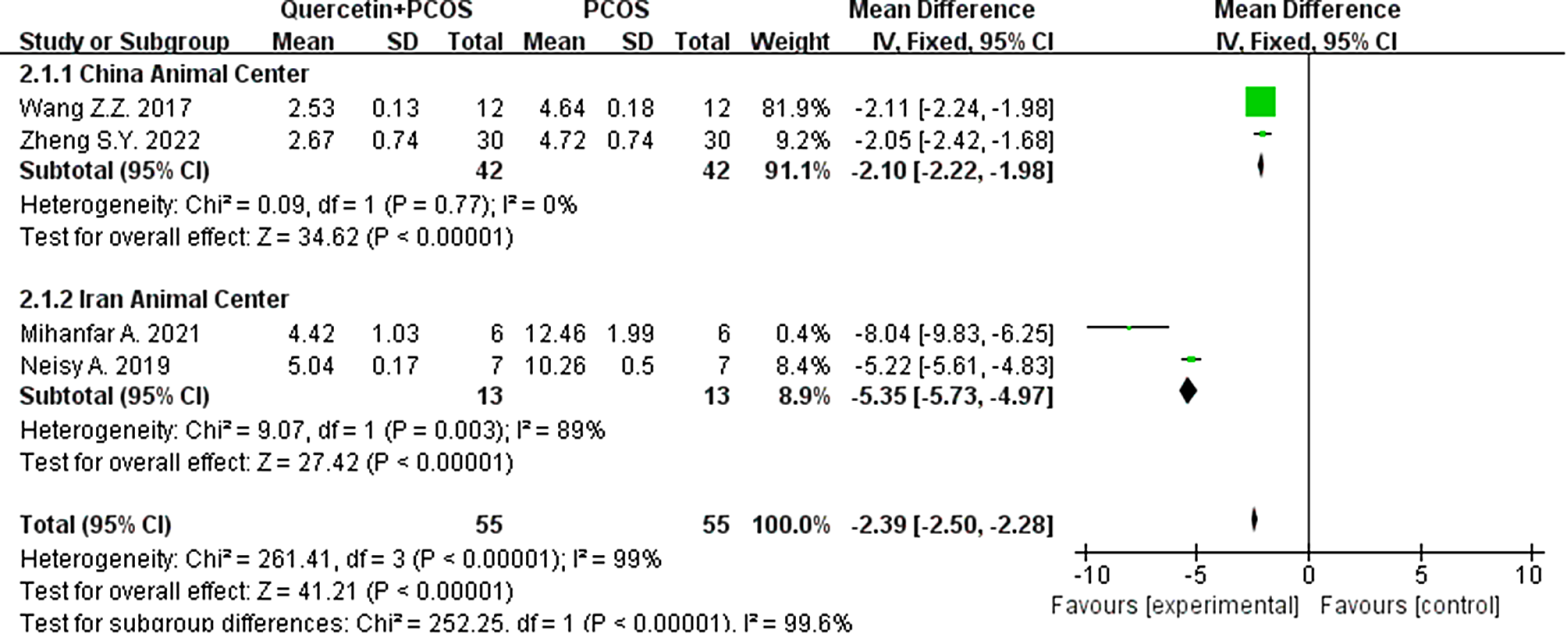
Forest plot of HOMA-IR divided into subgroups according to animal center in different countries

Subgroup analysis included animal centers in different countries as grouping criteria, and the results of the heterogeneity test showed that the subgroup I^2^ decreased to 89% when the animal center was in Iran and decreased to 0% when the animal center was in China. However, the results still support that quercetin can effectively reduce HOMA-IR in PCOS animals (Fig. 11).

#### Testosterone

**Fig. 12:**
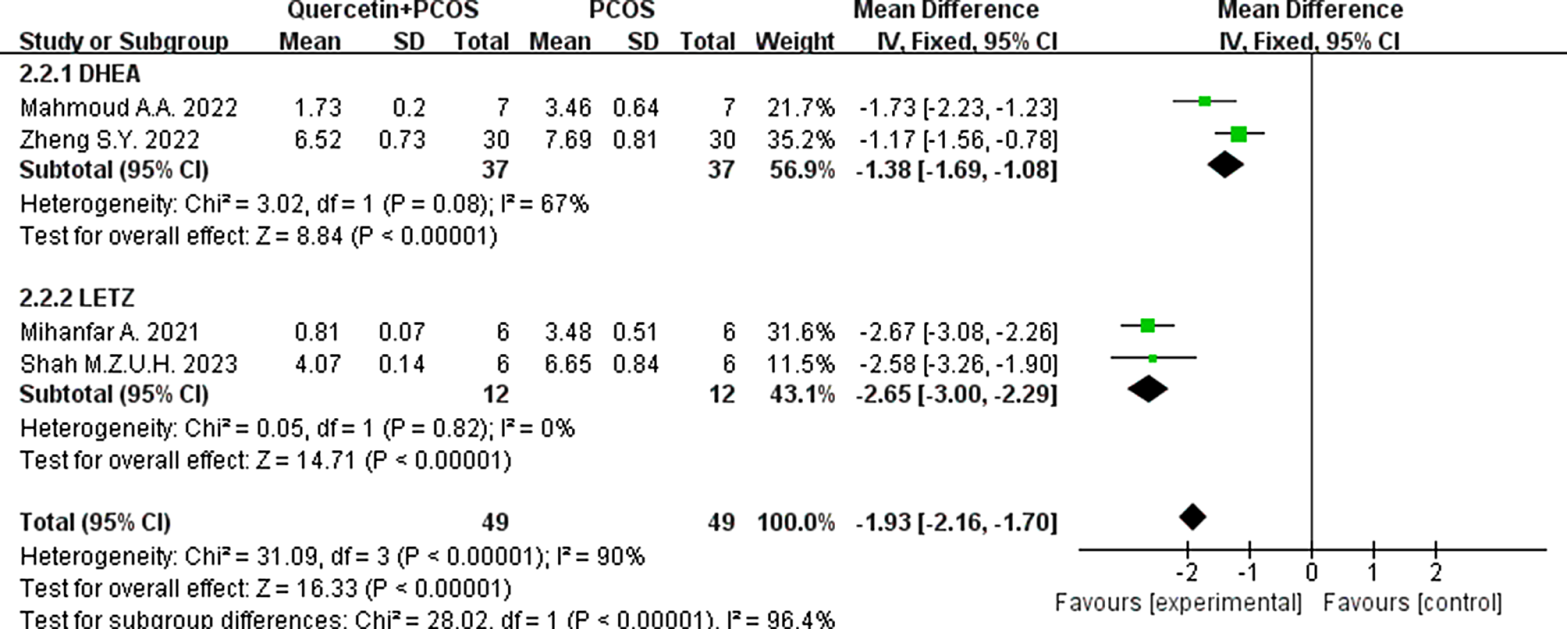
Forest plot of testosterone levels divided into subgroups according to modeling material

Subgroup analysis was used as a grouping criterion, and the results of the heterogeneity test showed that the subgroup I^2^ decreased to 67% when the rats were injected with DHEA to induce PCOS and decreased to 0% when the rats were given LETZ to induce PCOS. However, the results still support that quercetin can effectively reduce T levels in PCOS animals (Fig. 12).

### Publication bias

**Fig. 13:**
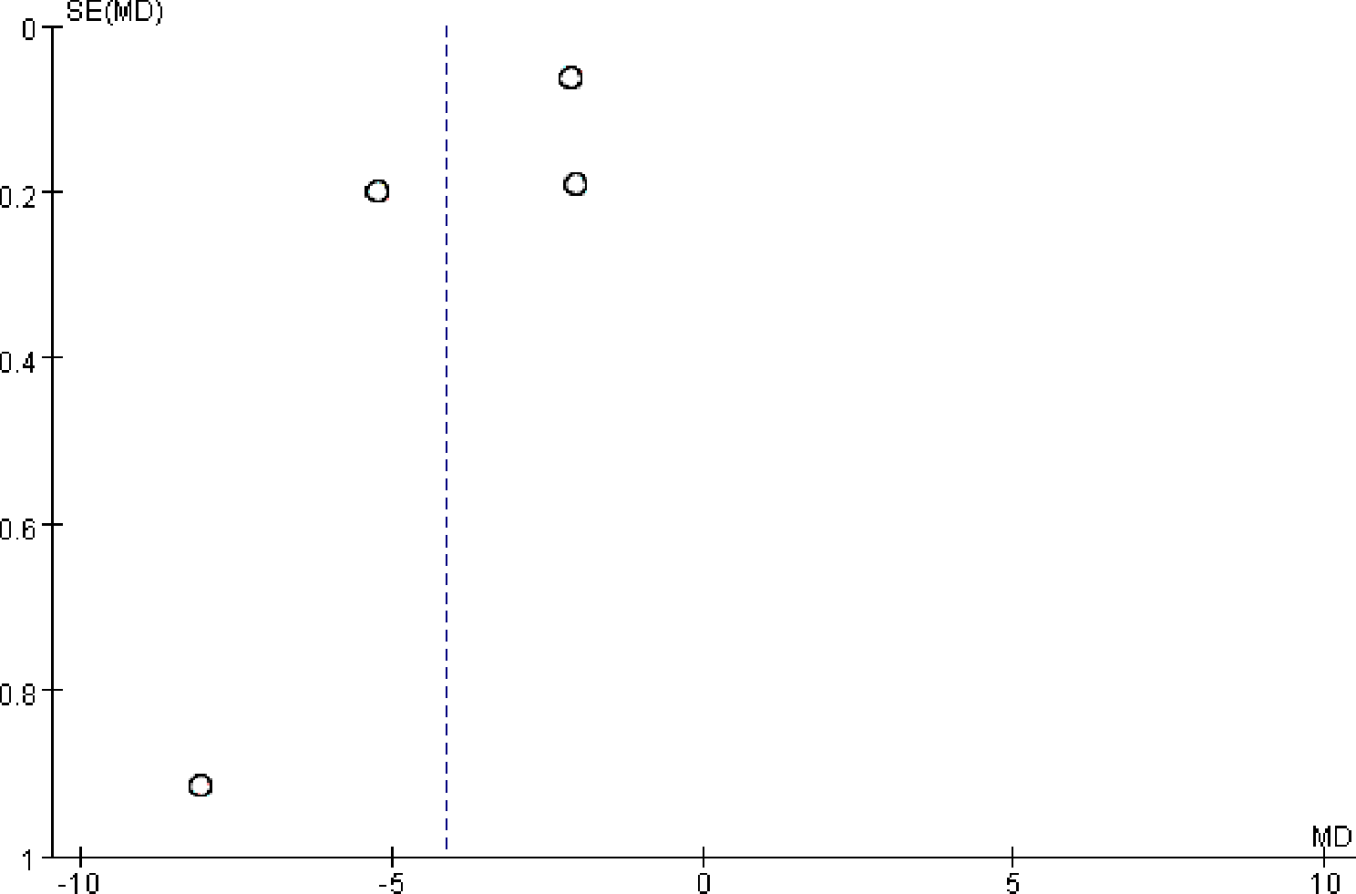
Funnel chart of HOMA-IR

The publication bias of HOMA-IR was assessed by a funnel plot. The results showed that there was a symmetrical trend and some publication bias (Fig. 13).

## Discussion

According to the present meta-analysis, quercetin has a beneficial effect on all aspects of PCOS. Quercetin significantly reduced FIS levels, FBG levels, HOMA-IR, cholesterol levels, TG levels, T levels, LH levels, LH/FSH ratios, VEGF levels and MDA levels in women with PCOS and increased SOD levels and the expression of GLUT4 mRNA compared with those in the model group. From the results of this study, it is worth mentioning that quercetin has been shown to positively treat PCOS through different biological aspects: (1) metabolic pathway: systematically indicated by insulin, blood sugar, cholesterol and TG. (2) Endocrine mechanisms, as observed for pituitary and ovarian hormones. (3) At the molecular level, GLUT4 gene expression and antioxidative stress are regulated.

Research has shown that insulin and blood sugar levels are elevated in PCOS rats during fasting [14]. Another study also indicated that GLUT4 is a very important insulin-dependent glucose transporter that mediates glucose transport in the uterus under insulin stimulation [23]. Research indicates that estrogen plays a pivotal role in modulating insulin sensitivity and preserving glucose homeostasis [24]. Through its two isoforms of receptors, ERα and ERβ, estrogen intricately regulates cell signaling. Ligands for ERα promote GLUT4 expression, thus significantly enhancing glucose tolerance. Conversely, ERβ ligands tend to counteract the actions of ERα, attenuating glucose uptake efficiency by inhibiting GLUT4 activity, potentially disrupting glucose homeostasis [25]. While consensus is yet to be reached regarding whether all ERβ ligands exhibit pro-diabetic characteristics, current investigations underscore a plausible link between ERβ ligands and abnormalities in glucose metabolism. The study’s findings reveal a diminished expression of uterine ERα in PCOS, potentially contributing to reduced GLUT4 expression exacerbated by diminished uterine estrogen activity in PCOS [14]. Quercetin emerges as a potential therapeutic candidate for alleviating insulin resistance in PCOS, as it may upregulate GLUT4 mRNA expression by enhancing the expression of ERα ligands. This mechanism suggests quercetin’s capacity to mitigate insulin resistance and regulate blood glucose levels in PCOS rats [14], indicating its therapeutic promise in addressing insulin resistance (IR).

Cytochrome P450c17, a pivotal enzyme within the androgen synthesis pathway, is encoded by the CYP17A1 gene [26] and exhibits dual functionalities as both a 17-hydroxylase and 17,20-lyase enzyme [27]. These enzymatic activities play a crucial role in augmenting androgen synthesis within granulosa cells, a process closely linked to the pathogenesis of PCOS. Consequently, quercetin shows promise in modulating androgen levels by regulating the activity of cytochrome P450c17. Moreover, research conducted by Shah et al. highlights quercetin’s anti-androgenic potential, elucidating its ability to effectively block the phosphatidylinositide 3-kinase (PI3K) inhibition pathway and down-regulate the CYP17A1 gene [28]. These findings underscore quercetin’s potential therapeutic utility in mitigating androgen-related abnormalities, particularly in the context of conditions such as PCOS.

According to research, PCOS rats also express elevated levels of androgen [19], which increases lipolysis in adipocytes induced by catecholamines, resulting in elevated levels of serum free fatty acids and abnormal blood lipids [29]. Further studies have shown that increased levels of androgen also lead to fat accumulation [30]. Adipose tissue serves not only as an energy reservoir but also as an active endocrine organ that orchestrates the inflammatory response and metabolic equilibrium of the body through the secretion of various biologically active adipokines, including adiponectin, leptin, and resistin. These pro-inflammatory adipokines exert a profound influence on the intricate physiological and pathological manifestations observed in patients with PCOS. Emerging research suggests that quercetin augments its anti-inflammatory and insulin-sensitizing properties by upregulating adiponectin expression and secretion, while concurrently downregulating the levels of resistin and leptin, two prominent pro-inflammatory mediators, within the adipose tissue of PCOS-afflicted rats. This modulation not only mitigates inflammation and enhances insulin sensitivity but also potentially curtails excessive androgen synthesis indirectly [19]. Additionally, quercetin treatment has been associated with a notable reduction in cholesterol and triglyceride levels, further underscoring its therapeutic potential in PCOS management. The application of quercetin successfully improved the abnormal blood lipids, blood glucose, and IR caused by PCOS, and its effect was similar to that of the standard positive therapeutic drug metformin. Therefore, these studies show that in an optimized PCOS model, there is no significant difference between quercetin and metformin in the treatment of IR in PCOS rats.

Experiments have shown that PCOS rats typically exhibit elevated levels of LH/FSH in serum [18]. In PCOS patients, excessive elevation of LH can inhibit the synthesis and secretion of FSH, leading to reduced FSH secretion in the body. This abnormal increase in LH/FSH is caused by endocrine abnormalities, disrupting the hypothalamic-pituitary-ovarian axis and hindering the development of ovarian granulosa cells, which is a significant cause of abnormal follicular growth and development. Studies have shown that quercetin can significantly reduce LH levels and the LH/FSH ratio in PCOS rats. This suggests that the target of action of quercetin is the pituitary-ovarian axis, as it can reverse the abnormal levels and quantities of LH/FSH.

Oxidative stress is considered to be a potential activator of PCOS, whereby the in vivo antioxidative enzyme system of patients with PCOS may be disrupted [31]. SOD is an important antioxidase for maintaining oxidative balance in the body, and its concentration decreases in PCOS rats, while MDA levels reflect the extent of oxidative stress-induced cell membrane damage, and its concentration increases in PCOS rats [8]. Studies have shown that with the use of quercetin, the concentration of SOD increases and that of MDA decreases in PCOS rats, which indicates that quercetin can prevent oxidative stress in PCOS rats.

Oxidative stress can induce inflammation, which is related to the development of PCOS [32]. Women with PCOS can release more VEGF, which may be related to the presence of androgen receptor binding sites in the VEGF promoter region [33]. When androgens bind to these sites, they trigger the expression of the VEGF gene, leading to increased production of VEGF [34]. When mice were treated with quercetin, the level of VEGF decreased. Furthermore, Zheng et al.‘s investigation demonstrated that quercetin administration in DHEA-induced PCOS model rats markedly reduces the expression levels of inflammatory markers such as IL-1β, IL-6, and TNF-α within ovarian tissues, indicating the notable anti-inflammatory efficacy of quercetin [16]. Moreover, Wang et al.‘s study elucidated the impact of quercetin on the inflammatory transcription factor NF-kB. Their findings revealed a significant inhibition of NF-kB nuclear translocation in granulosa cells of insulin-resistant PCOS rat models following quercetin treatment. This inhibitory effect coincided with the downregulation of various inflammation-related gene expressions in ovarian tissues, including nicotinamide adenine dinucleotide phosphate oxidase subunit p22phox, oxidized low-density lipoprotein, and Toll-like receptor 4 [17]. Collectively, these research findings provide compelling evidence of quercetin’s ability to attenuate inflammation through diverse pathways, thereby enhancing insulin sensitivity in individuals with PCOS.

An extensive examination of the underlying mechanisms underscores the pivotal roles of 5′AMP-activated protein kinase (AMPK) and Sirtuin-1 (SIRT-1) as metabolic regulators governing lipid and adiposity metabolism, the glycolytic process, and the response to oxidative stress. Mihanfar et al.‘s investigation elucidates that following quercetin treatment, there is a significant upregulation in the protein expression levels of p-AMPK and SIRT-1 within ovarian tissues of PCOS-afflicted rats, concomitant with heightened insulin sensitivity [19]. This finding suggests that quercetin potentially augments insulin signaling efficacy and mitigates insulin resistance through the activation of the AMPK-SIRT-1 signaling cascade. In the exploration of hepatic glucose metabolism imbalance, Neisy et al. observed a reduction in glucokinase (GK) activity alongside an elevation in hexokinase (HK) activity within the liver under conditions of PCOS. These alterations imply that the increased HK activity in the liver may serve as a compensatory mechanism for the diminished GK activity during IR. Quercetin may indirectly modulate and ameliorate hepatic HK and GK activity by enhancing insulin sensitivity, thereby facilitating the restoration of normal glucose homeostasis in the liver [14]. The dysregulation in the expression of apoptotic regulatory proteins, B-cell lymphoma 2 (Bcl-2) and Bcl-2-associated X protein (Bax), is recognized as a pivotal contributor to granulosa cell apoptosis in individuals with PCOS [35]. The study conducted by Zheng et al. revealed that administration of quercetin in a rat model of PCOS led to a significant downregulation of the expression levels of the pro-apoptotic protein Bax in ovarian tissue, alongside a notable enhancement in the expression of the anti-apoptotic protein Bcl-2 [16]. This dual modulation not only underscores the pivotal role of quercetin in governing cellular survival and apoptosis but also implies its potential to mitigate excessive granulosa cell apoptosis by reinstating an appropriate Bax/Bcl-2 ratio, thereby preserving ovarian function stability. Furthermore, Mahmoud et al.‘s investigation demonstrated that quercetin facilitates an increase in the abundance of Bcl-2 protein in ovarian tissue, thereby bolstering its protective effect on cell survival. Simultaneously, quercetin inhibits the abundance of Bax protein and the Bax/Bcl-2 ratio, a critical biological marker for curtailing follicular atresia and cystogenesis [18]. These findings collectively highlight the substantial promise of quercetin in ameliorating reproductive endocrine disorders associated with PCOS by intricately modulating the expression of Bcl-2 and Bax, thereby maintaining the dynamic equilibrium of ovarian follicles and suppressing pathological apoptosis processes.

### Shortcomings and prospects

The inclusion of a limited sample calls for further research to strengthen the discussion. Future studies need to further investigate more appropriate administration concentrations, timings, and treatment durations. Additionally, there is still no clear consensus on the use of appropriate animal models and the selection of suitable PCOS inducers. Finding animal models that mimic the pathogenesis of PCOS in humans remains an important research direction in PCOS animal experiments. However, considering the ethical limitations and complexities of human studies, animal models are an important resource for understanding PCOS characteristics and potential treatment methods. In a randomized clinical trial, researchers investigated the impact of quercetin on adiponectin-mediated insulin sensitivity among patients with PCOS [36]. The primary objective of the study was to assess quercetin’s potential influence on the endocrine dysfunction commonly associated with PCOS. Additionally, another randomized clinical trial examined the effects of quercetin on inflammation, hormone parameters, and pregnancy outcomes in women with PCOS [37]. These trials collectively offer valuable insights into the potential therapeutic benefits of quercetin in the management of PCOS. Nonetheless, further animal experiments are warranted to elucidate the precise mechanisms by which quercetin exerts its effects on PCOS and to ascertain its long-term safety and efficacy. In summary, quercetin emerges as a promising therapeutic agent with broad therapeutic applications, demonstrating encouraging efficacy in mitigating PCOS and its associated complications. Future investigations should prioritize advancing our understanding of the specific molecular pathways through which quercetin operates within the pathological milieu of PCOS. Moreover, proactive exploration of quercetin’s potential as a novel, targeted therapeutic strategy for endocrine disorders is imperative to furnish more precise and efficacious treatment modalities for the multitude of PCOS patients worldwide.

## Conclusion

In conclusion, this study demonstrated that quercetin has significant therapeutic effects in treating PCOS. Quercetin can exert its effects through the following pathways. In metabolic pathways, quercetin can systematically decrease insulin, blood glucose, cholesterol, and triglyceride levels. A reduction in these indicators can help adjust the metabolic environment in patients and alleviate PCOS. In endocrine pathways, quercetin can regulate the function of the pituitary-ovarian axis, decrease T and LH levels, and reduce the LH/FSH ratio. This regulatory effect helps restore normal endocrine levels in patients and improves PCOS. At the molecular level, quercetin can also regulate the expression of the GLUT4 gene, which is one of the key genes involved in glucose metabolism. Quercetin also has antioxidative effects, which can help alleviate oxidative stress damage caused by PCOS. Therefore, quercetin has great potential in the treatment of PCOS.

### Electronic supplementary material

Below is the link to the electronic supplementary material.

Supplementary Material 1

## Data Availability

The datasets for this study are within the paper.
